# LncRNAs in Stem Cells

**DOI:** 10.1155/2016/2681925

**Published:** 2016-01-06

**Authors:** Shanshan Hu, Ge Shan

**Affiliations:** School of Life Sciences and CAS Key Laboratory of Brain Function and Disease, University of Science and Technology of China, Hefei, Anhui 230027, China

## Abstract

Noncoding RNAs are critical regulatory factors in essentially all forms of life. Stem cells occupy a special position in cell biology and Biomedicine, and emerging results show that multiple ncRNAs play essential roles in stem cells. We discuss some of the known ncRNAs in stem cells such as embryonic stem cells, induced pluripotent stem cells, mesenchymal stem cells, adult stem cells, and cancer stem cells with a focus on long ncRNAs. Roles and functional mechanisms of these lncRNAs are summarized, and insights into current and future studies are presented.

## 1. Introduction

Less than 2% of human genome is composed of protein-coding genes, but transcription is widespread and corresponding to more than ~80% of the genome [1, 2]. With the development of deep sequencing and bioinformatics, a large number of noncoding RNAs (ncRNAs) are identified in eukaryotic cells [3, 4]. NcRNAs greater than 200 nucleotides are grouped as long noncoding RNAs. Majority of lncRNAs are intergenic transcripts and sense or antisense to other transcripts. Similar to mRNAs, most lncRNAs are Pol II transcripts which have a poly-A tail and 5′ cap. But compared to protein-coding genes, lncRNAs show lower evolutionary conservation and lower expression level. LncRNAs are predominantly localized in nucleus [5–7]. LncRNAs have been associated with significant roles in diverse biological events such as chromatin modifications, transcriptional regulation, and posttranscriptional regulation [8–10].

Stem cells are undifferentiated cells that can either self-renew or differentiate into specialized cells. Stem cells are of great interest to both basic and biomedical research due to their unique properties in cell biology [11–13]. In recent years, researchers are paying more and more attention to roles of ncRNAs in stem cells.

In this review, we will discuss some of the known roles of ncRNAs in stem cells, including embryonic stem cells (ESCs), induced pluripotent stem cells (iPSCs), mesenchymal stem cells (MSCs), adult stem cells, and cancer stem cells.  Table 1 gives a brief summary to key properties of each stem cell type. The focus is on lncRNAs, and for roles of small ncRNAs such as microRNAs and piRNAs in stem cells, readers are referred to other articles [14–17].

## 2. LncRNAs in ESCs

Mohamed et al. performed a genome-wide screen to identify lncRNAs transcriptionally regulated by Oct4 and Nanog in mouse ESCs (mESCs) by combining the mESC transcriptome with the chromatin immunoprecipitation genomic location maps of Oct4 and Nanog [18]. Two conserved lncRNAs AK028326 and AK141205 were further confirmed to be directly activated by Oct4 or suppressed by Nanog, respectively. In fact, AK028326 might be a coactivator of Oct4. Later, a systematic survey for lincRNAs, which are a subclass of lncRNAs transcribed from intergenic regions of coding genes, was conducted with several murine cell lines including ESCs [19]. More than a thousand lincRNAs were identified, and many of them might possess putative functions in the pluripotency or proliferation of ESCs. Transcription of some lincRNAs was also regulated by key transcription factors such as Sox2, Oct4, and Nanog in ESCs.

In human ESCs (hESCs), multiple ESC specific lncRNAs were identified with microarray assays [20]. Some of these lncRNAs were involved in pluripotency maintenance and interacted with SOX2 and SUZ12 (a subunit of PRC2 complex). A subset of the identified lncRNAs was involved in neurogenesis of ESCs, and these lncRNAs potentially played neurogenic roles via physical interaction with important epigenetic factors such as REST and SUZ12. One of the lncRNAs identified in this study, rhabdomyosarcoma 2-associated transcript (RMST), was further investigated in a later study [21, 22]. RMST interacted directly with and served as a transcriptional coregulator of SOX2 to modulate the transcription of a number of SOX2 downstream genes. Recently, the long terminal repeats of human specific endogenous retrovirus subfamily H (HERVH), which is transposable elements expressed preferentially in hESCs, was found to function as nuclear lncRNAs with associations with OCT4, coactivators such as p300 and Mediator subunits [23].

A recent report showed that pluripotency-associated transcription factor SOX2 has an overlapping long noncoding transcript SOX2OT, which was highly expressed in embryonic stem cells and downregulated upon the induction of differentiation [24]. SOX2OT had a potential role in modulating pluripotency through the regulation of* SOX2* expression.

Another lincRNA, linc-RoR might function as microRNA sponge to prevent mRNA of some key transcription factors in hESCs from microRNA mediated regulation [25]. Thus, this lncRNA played a role in ESC maintenance and differentiation in the cytoplasm.

These results implicated that lncRNAs could modulate the pluripotency and differentiation of ESCs via intervening the transcriptional and epigenetic regulatory networks in the nucleus or tuning the microRNA functions in the cytoplasm. Other cytoplasmic and nuclear roles of lncRNAs in ESCs are waiting for further investigations.

## 3. LncRNAs in iPSCs

The conversion of lineage-committed cells to iPSCs is a process called reprogramming. In a 2010 paper, Rinn Lab characterized the transcriptional reorganization of lincRNAs and identified a vast number of lincRNAs associating with reprogramming of human iPSCs (hiPSCs) [26]. These lincRNAs were overexpressed in both hESCs and hiPSCs but showed an elevated level in iPSCs compared to ESCs, suggesting that their activation might promote the iPSCs. One of the lincRNAs named lincRNA-RoR for “regulator of reprogramming” was regulated by pluripotency transcription factors such as OCT4, SOX2, and NANOG. Knockdown of lincRNA-RoR resulted in an inhibition in reprogramming. Interestingly, linc-RoR was later found to function as a competing endogenous RNA in hESCs as already discussed.

By analyzing single-cell transcriptomes during somatic cell reprogramming, more than 400 hundred lncRNAs were characterized that were dynamically expressed at defined stages of reprogramming [27]. During reprogramming, sets of relevant lncRNAs were activated to modulate signaling pathways, repress lineage-specific genes, and regulate metabolic gene expression.

The reprogramming process is accompanied by global epigenetic remodeling [28]. The lncRNA* Xist* responsible for X chromosome inactivation (XCI) has been investigated as a classic epigenetic regulator in the context of reprogramming [29, 30]. Female mESCs have low* Xist* expression and two active X chromosomes. Successfully reprogrammed female murine iPSCs (miPSCs) have undergone X chromosome reactivation and have the same feature in* Xist* expression as mESCs. Upon differentiation, female mESCs and miPSCs initiate* Xist* expression and XCI. Surprisingly, hESCs and hiPSCs can be divided into three classes based on their different states of* Xist* expression. Class I hESCs and hiPSCs behave just like mESCs and miPSCs. Class II human pluripotent stem cells express* Xist* and exhibit random XCI even before differentiation. Class III cells have lost* XIST* expression but have already initiated XCI, regardless of whether they are in the undifferentiated or differentiating conditions [31].

It seems that lncRNAs play essential roles in the reprogramming process. A better understanding in lncRNAs and more broadly ncRNAs in the context of iPSCs would certainly benefit future biomedical research aiming to utilize iPSCs in the clinic.

## 4. LncRNAs in MSCs and the Mesodermal Lineage

Mesenchyme is connective tissue derived from the mesoderm during animal development. MSCs are first identified in the bone marrow (BM) stroma. BM MSCs provide microenvironment for hematopoietic stem cells and can also differentiate into various mesodermal lineages. Later, MSCs are found in many tissues such as placenta, umbilical cord blood, adipose tissue, muscle, corneal stroma, and the dental pulp of deciduous baby teeth. These MSCs with different origins all possess property to differentiate into mesodermal lineages. Thus, MSCs are an important source of multipotent cells with great potential of clinic applications. LncRNAs in MSCs have significant regulatory roles and potentially can be used as biomarkers for disease or therapeutic targets. Wang et al. performed differential expression profiles of lncRNAs and mRNAs of undifferentiated versus chondrodifferentiated human BM MSCs using microarray in 2015 [32]. Some of the identified lncRNAs could be important regulators in chondrogenic differentiation. The same group identified lncRNAs with differential expression and putative functions during the osteogenic differentiation of human BM MSCs [33]. A total of 1,206 differentially expressed lncRNAs were identified, and among them 687 were upregulated and 519 downregulated, more than twofold.

Some lncRNAs are functionally required during adipogenesis [34]. Brown adipose tissue (BAT) and white adipose tissue (WAT) are differentiated from MSCs in adipose tissues. Sun et al. performed RNA sequencing and identified 175 lncRNAs in total from* in vitro* cultured brown and white preadipocytes,* in vitro* differentiated mature brown and white adipocytes, and primary brown and white mature adipocytes directly isolated from mice [34]. These lncRNAs were up- or downregulated during differentiation of both brown and white adipocytes. Activation of some transcriptional factors and cofactors such as peroxisome proliferator-activated receptor *γ* (PPAR*γ*) and CCAAT/enhancer-binding protein *α* (CEBP*α*) is the hallmark of brown adipocyte differentiation. These lncRNAs have promoters with binding of PPAR*γ* and/or CEBP*α* key transcription factors. RNAi-mediated loss-of-function identified the top 20 lncRNAs that may be involved in the proper differentiation of adipocyte precursors. Later in 2014, substantially more lncRNAs were identified and may be functionally required during proper adipogenesis [35]. Zhao and colleagues identified inducible lncRNAs involved in brown fat development and thermogenesis using transcriptional profiling in adipose tissues and cells during brown adipocyte differentiation [36]. Twenty-one lncRNAs were found to be enriched in BAT, and among them AK038898 significantly impaired brown adipogenesis upon RNAi knocking down. This lncRNA was then renamed brown fat lncRNA 1 (Blnc1). The expression of Blnc1 was highly induced during brown and beige adipogenesis. The induction was correlated with the stimulation of the thermogenic gene program and in consistency with the expression of key thermogenic markers such as Ucp1 in adipose tissues. Blnc1 is primarily localized in the nucleus and forms a ribonucleoprotein complex with EBF2, which is a member of the EBF family of transcription factors that has been implicated in the control of adipogenesis [37]. Blnc1 itself is also a target of EBF2, thereby forming a feed-forward regulatory loop to regulate adipocyte gene expression. Knowing the distinct roles and functional mechanisms of lncRNAs in adipogenesis have great values in developing new approaches to fight obesity and other related pathological consequences.

LncRNAs also regulate cardiomyocyte differentiation. Zhu et al. identified multiple lncRNAs involved in the development of the lateral mesoderm and in the differentiation of cardiomyocytes with P19 cells, which were isolated from embryo-derived mouse teratocarcinoma and could differentiate into cardiacmyocytes [38]. Forty differentially expressed lncRNAs, 28 upregulated and 12 downregulated, were identified using microarrays. In another research, Matkovich and colleagues used genome-wide sequencing and improved bioinformatics to measure dynamic lncRNA regulation during the transition between embryo and adult mouse hearts and identified numerous cardiac-specific lncRNAs [39]. Analyses indicated a broad role of regulated cardiac lncRNAs as modulators of key cardiac transcriptional pathways. Two lncRNAs, Fendrr and Braveheart, were later found to be involved in the development of the lateral mesoderm in the heart and the differentiation of cardiac myocytes, respectively [40, 41]. Fendrr was discovered in a survey of differentially expressed lncRNAs in six different tissues dissected from early somite-stage of mouse embryos by using RNA-seq and ChIP-seq [40]. Knocking out Fendrr by homologous recombination was embryonic lethal. The ChIP data showed that Fendrr interacts directly with the PRC2 and/or TrxG/MLL complexes in mouse embryos, indicating that it acts as a modulator of chromatin signatures defining gene activity in embryonic development. Braveheart also plays roles as an epigenetic factor [41]. This lncRNA is required for the progression of nascent mesoderm towards a cardiac fate by interacting with SUZ12, a component of PRC2 to alter cardiomyocyte differentiation and retain the cardiac phenotype in neonatal cardiomyocyte [41]. Cardiovascular diseases are currently the main cause of morbidity worldwide, and researches on these lncRNAs are critical to achieving a better understanding of heart development and designing new ways for the diagnosis and treatment of cardiac related diseases.

Several important lncRNAs play critical roles in myogenesis [42]. Linc-MD1 is a muscle-specific long noncoding RNA expressed during early phases of muscle differentiation. It promotes the switch of muscle differentiation from early to later stages by acting as a miR-133 and miR-135 sponge [43]. What is more interesting is that linc-MD1 is also the primary transcript of miR-133b [44]. The process of linc-MD1 as a pri-microRNA by Drosha is controlled by HuR. The binding of HuR to linc-MD1 suppresses Drosha cleavage of linc-MD1 and leads to accumulation of linc-MD1, which further inhibits the functions of miR-133 as a sponge so that this microRNA could not target the HuR mRNA. Thus, HuR and linc-MD1 form an elegant feed-forward positive loop [45]. Another lncRNA YAM-1 affects myogenesis via epigenetic transcriptional activation [42]. The transcription factor Yin Yang 1 (YY1) regulates expression multiple genes in myoblasts, and YAM-1 is positively regulated by YY1. YAM-1 expression is downregulated during myogenesis and its expression inhibits myoblast differentiation. YAM-1 regulates the transcription of miR-715 in* cis* and miR-715 is a microRNA that targets Wnt7b, an important signaling modulator of myogenesis [42].

## 5. LncRNAs in Adult Stem Cells

A series of studies focused on lncRNAs in adult stem cells such as hematopoietic stem cells and adult neural stem cells. The milestone discovery of lincRNA HOTAIR in 2007 was carried out with primary human fibroblasts, which are a kind of somatic stem cell [45]. In this and following researches, HOTAIR was found to bind both the polycomb repressive complex 2 (PRC2) and the LSD1/CoREST/REST complex and thus to modulate histone modifications on target genes [45, 46]. The lncRNA ANCR (antidifferentiation noncoding RNA) was also characterized by epidermal progenitors versus differentiating cells [47]. Knocking down ANCR alone led to rapid expression of differentiation genes. ANCR was thought to be essential for the undifferentiated state, although the exact molecular mechanism requires further investigation [47]. On the other hand, an lncRNA called terminal differentiation-induced noncoding RNA (TINCR) promoted epidermal differentiation [48]. TINCR had a very low level in epidermal stem cells, but it was dramatically induced upon differentiation. As a cytoplasmic ncRNA, TINCR could interact with mRNAs of many differentiation genes such as KRT80 and RNA-binding protein STAU1 to mediate mRNA stabilization through binding TINCR box.

In a systematic analysis of adult neural stem cell lineage in the mouse subventricular zone for lncRNA with potential roles in adult neurogenesis, Ramos et al. identified two lncRNAs Six3os and Dlx1as playing crucial roles in the lineage specification of adult stem cells [49]. The same lab later characterized a neural-specific lncRNA named Pnky, which localized in the nucleus of human and mouse neural stem cells [50]. With knockdown of Pnky, neuronal differentiation increased both* in vitro* and* in vivo*. Pnky interacted with the splicing regulator PTBP1 and thus regulated the expression and alternative splicing.

Recently, Luo et al. identified 159 unannotated lncRNAs enriched in hematopoietic stem cells with RNA sequencing. Knocking down two of these lncRNAs showed effects on self-renewal and lineage commitment of hematopoietic stem cells [51]. Interestingly, the genomic binding sites of one lncRNA overlapped significantly with binding sites of a key hematopoietic transcription factor E2A, indicating this lncRNA might be a cofactor of the transcription factor.

It is obvious that lncRNAs possess vital roles in a variety of somatic stem cells with diverse mechanisms. Considering the large number of lncRNAs identified, we still have a long way to understand the full range of roles of these regulators in adult stem cells as well as in the other eukaryotic cells.

## 6. LncRNAs in Cancer Stem Cells

Cancer stem cells (CSCs) play critical roles in tumor initiation, progression, metastasis, chemoresistance, and recurrence [52, 53]. Some studies showed that lncRNAs might be involved in regulating stem cell signaling in CSCs.

A novel lncRNA HIF2PUT (hypoxia-inducible factor-2*α* promoter upstream transcript) was identified in a kind of CSCs, the osteosarcoma stem cells [54]. HIF2PUT expression levels were positively correlated with its parent gene* HIF-2α* in osteosarcoma tissues, indicating a regulatory role of HIF2PUT through HIF-2*α*. Knocking down HIF2PUT enhanced the proliferation, migration, and self-renewal of osteosarcoma stem cells while overexpression of HIF2PUT decreased the proliferation, migration, and self-renewal [54]. In liver CSCs, lncTCF7 was identified, and it recruited the SWI/SNF complex and further activated the Wnt signaling to promote self-renewal of liver CSCs and tumor propagation [55].

The lncRNA MALAT-1 (metastasis-associated lung adenocarcinoma transcript 1) was found to be upregulated in CSCs, and high expression levels of this lncRNA positively correlated with the proportion of CSCs in pancreatic cancer cells [10, 56, 57]. MALAT-1 might serve as an oncogenic lncRNA in pancreatic cancer by promoting epithelial-mesenchymal transition and stimulating the expression of self-renewal factors such as Sox2.

An lncRNA H19 known for more than two decades also functions in cancer and cancer stem cells [58, 59]. H19 is an imprinting gene in both mice and human [60, 61]. H19 RNA was identified as a tumor suppressor and was involved in Wilms' tumor long time ago in early 1990s [62, 63]. Despite the fact that H19 is one of the first identified, most abundant and conserved ncRNAs in mammals, its actually physiological functions and functional mechanism have been elusive for a long time. In 2007 and then in 2012, compelling lines of evidence showed that H19 is the primary transcript of a microRNA miR-675 [64, 65]. Overexpression of miR-675 in embryonic and extraembryonic cell lines reduced proliferation. Just like the linc-MD1 (the primary transcript of miR-133b), the processing of H19 into miR-675 is also regulated by HuR. What makes H19 more intriguing is the finding that H19 could be sponge of let-7 and miR-106a (a miR-17-5p family member) [66, 67]. HOTAIR with high expression level was always involved in various cancers as well as CSCs via promoting tumorigenesis, cell proliferation, or tumor metastasis and could be a novel epigenetic molecular target for therapeutics [68, 69]. Linc00617 may be another potential therapeutic target for breast cancer because of functioning as a key regulator of EMT and promotes cancer progression and metastasis via activating the transcription of Sox2 [70].

Knowing the roles of lncRNAs in tumor cells and especially in CSCs would help the development of tumor diagnosis and therapeutics [71]. So far, lncRNA research in cancer and CSCs is just emerging, and more efforts are required to push the field forward.  Table 2 lists lncRNAs, their regulatory roles, and stem cells where they exert functions.

## 7. Conclusions and Perspectives

**Figure 1:**
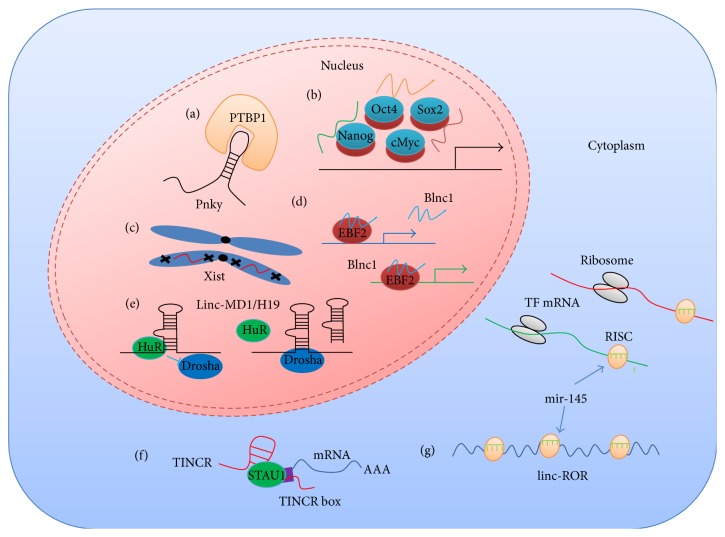
Some known functional mechanisms of lncRNAs in stem cells. (a) Pnky localizes to the nucleus and interacts with the mRNA splicing regulator PTBP1 to regulate splicing of key transcripts; (b) some lncRNAs serve as cofactors of key transcription factors (TF) to regulate gene transcription; (c) Xist as an epigenetic regulator of X chromosome inactivation plays roles in female ESCs and iPSCs; (d) Blnc1 is activated by EBF2 and functions as a coactivator of EBF2; (e) linc-MD1 is the primary transcript of miR-133b and H19 is the primary transcript of miR-675, and the Drosha processing of both is regulated by HuR; (f) TINCR interacts with STAU1 and multiple mRNAs via an RNA motif called TINCR box; (g) linc-ROR functions as a mir-145 sponge.

LncRNAs and other ncRNAs play substantial roles in diverse stem cell types. Here we have summarized some of the lncRNAs in five classes of stem cells, although we are not trying to summarize all the known lncRNAs in all kinds of stem cells and have to miss a lot of researches about lncRNAs in multiple other somatic and germline stem cells. LncRNAs could participate in the biology of stem cells with diverse mechanisms by serving as transcriptional coactivator or corepressor, modular scaffold for chromatin modifiers, factors of tuning splicing, primary transcripts of microRNAs, competing endogenous RNAs of microRNAs, and so on (Figure 1). We need to keep in mind that the functions and mechanisms of a vast majority of lncRNAs in stem cells remain unknown. The pervasive transcription of the genome and the growing inventory of ncRNAs with newly identified ncRNAs such as circRNAs and EIciRNAs also make a call for enormous future efforts to investigate ncRNAs in stem cells [72–77]. Nevertheless, the field of ncRNA research in stem cells has emerged and has been making progress towards a systematic understanding of stem cell biology. Researches in this area would eventually bring benefit to the understanding of lncRNAs and Biomedicine.

## Figures and Tables

**Table 1:** Key properties of each stem cell type.

Stem cell	Key properties
Embryonic stem cell	Derived from the blastocyst stage early mammalian embryos and has the ability to differentiate into any cell type and propagate

Induced pluripotent stem cell	A type of pluripotent stem cell that can be generated directly from adult cells; classic four specific genes encoding transcription factors (Oct4, Sox2, cMyc, and Klf) could convert adult cells into iPSCs, holding great promise in the field of regenerative medicine because of indefinite propagation

Mesenchymal stem cell	Multipotent stromal cells that can differentiate into a variety of cell types including osteoblasts, chondrocytes, myocytes, and adipocytes

Adult stem cell	Undifferentiated cells, which are found throughout the body after development, can divide or self-renew indefinitely, and generate all the cell types of the organ from which they originate

Cancer stem cell	Cancer cells that possess the ability to give rise to all cell types found in particular cancer samples and can generate tumors through the stem cell processes of self-renewal and differentiation into multiple cell types

**Table 2:** Regulatory roles of lncRNAs in different stem cells.

LncRNA	Stem cell types	Regulations and functions
AK028326	ESCs	Transcriptionally regulated by Oct4 and Nanog

AK141205	ESCs	A coactivator of Oct4

RMST	ESCs	Involved in pluripotency maintenance and interacting with SOX2 and SUZ12

HERVH	ESCs	Function as nuclear lncRNAs with associations with OCT4, coactivators such as p300 and Mediator subunits

SOX2OT	ESCs	Modulate pluripotency through the regulation of SOX2 expression

Linc-RoR	ESCs, iPSCs	Function as microRNA sponge to prevent mRNA of some key transcription factors

Xist	ESCs, iPSCs	An epigenetic regulator of X chromosome inactivation

Blnc1	MSCs	Highly induced during brown and beige adipogenesis and form a feed-forward regulatory loop with EBF2 to regulate adipocyte gene expression

Fendrr	MSCs	Interact directly with the PRC2 and/or TrxG/MLL complexes and act as a modulator of chromatin signatures defining gene activity in embryonic development

Braveheart	MSCs	An epigenetic factor, required for the progression of nascent mesoderm towards a cardiac fate by interacting with SUZ12

Linc-MD1	MSCs	Promote the switch of muscle differentiation from early to later stages by acting as miR-133 and miR-135 sponge

YAM-1	MSCs	Affect myogenesis via epigenetic transcriptional activation

HOTAIR	ASCs, CSCs	Bind both the polycomb repressive complex 2 (PRC2) and the LSD1/CoREST/REST complex and thus modulate histone modifications on target genes

ANCR	ASCs	Essential for the undifferentiated state

TINCR	ASCs	Interact with mRNAs of many differentiation genes such as KRT80 and RNA-binding protein STAU1 to mediate mRNA stabilization through binding TINCR box

Six3os	ASCs	Play crucial roles in the lineage specification of adult stem cells

Dlx1as	ASCs	Play crucial roles in the lineage specification of adult stem cells

Pnky	ASCs	Interact with the splicing regulator PTBP1 and regulate the expression and alternative splicing

HIF2PUT	CSCs	A regulatory role through HIF-2*α*

lncTCF7	CSCs	Recruit the SWI/SNF complex and further activated the Wnt signaling to promote self-renewal of liver CSCs and tumor propagation

MALAT-1	CSCs	Serve as an oncogenic lncRNA in pancreatic cancer by promoting epithelial-mesenchymal transition and stimulating the expression of self-renewal factors such as Sox2

H19	CSCs	Primary transcript of a microRNA miR-675; processing of H19 into miR-675 is also regulated by HuR

Linc00617	CSCs	Function as a key regulator of EMT and promote cancer progression and metastasis via activating the transcription of Sox2
